# Methodological fragmentation and integration priorities in psychedelic-assisted therapy assessment: a scoping review

**DOI:** 10.3389/fpsyt.2026.1880744

**Published:** 2026-08-06

**Authors:** Maria Laura Mele, Stefano Federici

**Affiliations:** 1 Myèsis Insight Center, Center for Research and Psychotherapy, Rome, Italy; 2 Società Italiana di Medicina Psichedelica (SIMEPSI), Italian Society for Psychedelic Medicine, Bari, Italy; 3 Department of Philosophy, Social and Human Sciences and Education, University of Perugia, Perugia, Italy

**Keywords:** assessment methods, implementation science, outcome measurement, psychedelic-assisted psychotherapy, psychedelic-assisted therapy, psychotherapy process, scoping review

## Abstract

**Introduction:**

Psychedelic-assisted therapy (PAT) is an emerging complex intervention for psychiatric and existential conditions (i.e., forms of distress related to meaning, mortality, and identity, such as that arising in life-threatening illness); however, a systematic characterization of the evaluation methods used across the entire clinical intervention remains incomplete. This scoping review synthesizes the scientific literature on the methodologies and frameworks for evaluating PAT, covering pre-session screening, acute experience, intervention processes, post-session integration, clinical and existential outcomes, safety, and service evaluation.

**Methods:**

Following PRISMA-ScR (Preferred Reporting Items for Systematic Reviews and Meta-Analyses extension for Scoping Reviews) guidelines, we analyzed 59 peer-reviewed publications, comprising contemporary PAT literature published between 2008 and 2026 and foundational psychometric sources for established instruments used in PAT. The findings were categorized into four evaluation domains: (i) assessment instruments for subjective experiences; (ii) clinical intervention models and competency frameworks; (iii) clinical and existential outcome measures; and (iv) safety, ethics, and implementation frameworks.

**Results:**

The analysis reveals a number of advances, including the development and cross-cultural adaptation of validated instruments for measuring mystical and challenging experiences. Clinical intervention frameworks emphasizing the importance of the “therapeutic container” have been articulated, and preliminary work has begun to identify core practitioner competencies. PAT has been associated with preliminary findings from controlled studies for conditions such as depression, anxiety, post-traumatic stress disorder, and existential distress, although demographic representation in trials remains limited, and methodological constraints (e.g., small samples and challenges in blinding) warrant cautious interpretation. The review also identifies gaps: methodological integration is still incomplete, with different assessment domains rarely combined into unified protocols; measures for post-session integration and existential outcomes remain undervalidated; and safety monitoring and implementation science present areas requiring standardization.

**Discussion:**

This review maps the current methodological landscape, documenting areas of advancement and defining priorities for developing the integrated, multidimensional evaluation models required to advance the field.

## 1 Introduction

Psychedelic-assisted therapy (PAT) has re-emerged as a complex clinical intervention for psychiatric conditions and forms of existential distress related to meaning, mortality, and identity, particularly in the context of life-threatening illness (1, 2). Standard psychopharmacological models conceptualize the psychedelic substance as the sole active agent (3); however, PAT operates as a complex intervention in which clinical effects emerge from the interaction between the pharmacological action of the administered compound and the structured clinical intervention context (i.e., the protocolized sequence of preparation, dosing, and integration sessions within which the pharmacological agent is administered) (4). Contemporary clinical investigation has renewed systematic interest in the clinical use of classic serotonergic psychedelic compounds, i.e., psilocybin, lysergic acid diethylamide (LSD), mescaline, and N,N-dimethyltryptamine (N,N-DMT), which may be administered either as an isolated compound or as part of DMT-containing preparations such as ayahuasca, as well as the entactogen 3,4-methylenedioxymethamphetamine (MDMA). This review includes MDMA as it shares a structured clinical model with classic psychedelics, centered on a compound-induced nonordinary state embedded within preparatory and integrative therapy sessions (5). In contrast, ketamine-based interventions were excluded because ketamine possesses a distinct pharmacodynamic profile as an N-methyl-D-aspartate (NMDA) receptor antagonist and is typically embedded in clinical architectures with a different structure and phenomenological emphasis (4, 6).

The epistemological status of psychedelic interventions requires clarification. The literature often uses the terms “psychedelic-assisted therapy” (PAT) and “psychedelic-assisted psychotherapy” (PAP) interchangeably (7), although a conceptual distinction can be analytically proposed. PAT may be understood as a broad category of interventions in which psychological support is provided during the administration of a psychoactive compound, without necessarily being embedded within a fully specified psychotherapeutic model. In contrast, PAP typically refers to interventions that are explicitly grounded in established psychotherapeutic frameworks, where the psychedelic experience is integrated within a structured therapeutic process. Because psychedelic interventions combine a pharmacological trigger with structured psychological support, PAT and PAP are best viewed as points along a continuum of therapeutic structuring rather than as mutually exclusive categories. Despite these conceptual distinctions, the current literature predominantly employs the acronym “PAT” as an umbrella term encompassing a range of supportive and psychotherapeutic modalities. To maintain consistency with the reviewed corpus, this manuscript adopts the term “PAT” to denote the integration of psychedelic administration within a structured clinical framework.

Recent placebo-controlled trials and meta-analyses have reported symptom reductions, with some pooled between-group effect sizes characterized as large; however, comparisons with standard psychopharmacological treatments remain methodologically constrained (1). Such estimates, however, warrant cautious interpretation due to methodological constraints inherent to psychedelic research, including limited sample sizes, demographic underrepresentation, short follow-up periods, and persistent challenges in maintaining effective blinding (1, 3, 8, 9), all of which complicate the estimation of treatment effects. The methodological foundations of PAT research have developed in a fragmented manner, with the various methodologies and frameworks employed for assessment and monitoring having evolved as parallel, domain-specific systems, without convergence into integrated frameworks. This fragmentation complicates efforts to establish standardized, replicable protocols for research and clinical practice.

The conceptualization of PAT as a complex intervention necessitates multidimensional assessment frameworks designed to evaluate the interaction between neurochemical mechanisms and clinical intervention processes, extending beyond conventional symptom reduction metrics (3). The clinical and methodological assessment of PAT is multidimensional, encompassing distinct phases of the clinical intervention: pre-session screening and preparation, acute experience assessment, therapeutic process evaluation, post-session integration assessment, clinical and existential outcome measurement, and safety monitoring (10). Although the theoretical understanding of PAT’s mechanisms has advanced (11), the translation of these models into validated and integrated assessment protocols remains a gap in the literature.

The evaluation of PAT is intrinsically linked to the theoretical models guiding the intervention (10, 12). The literature identifies a spectrum of psychotherapeutic frameworks applied within PAT, ranging from nondirective approaches to structured cognitive and somatic modalities (10). These frameworks propose partially overlapping mechanisms of change, including meaning-making processes, emotional breakthrough, cognitive integration, and relational dynamics. Accordingly, different assessment domains have been prioritized across studies. Models emphasizing experiential and meaning-making processes tend to foreground the measurement of acute subjective phenomena (e.g., mystical or challenging experiences) (10, 13, 14), whereas approaches focusing on integration and psychological change often incorporate measures of psychological flexibility, symptom reduction, and relational processes (10, 12, 13). However, in practice, many assessment instruments are used across multiple theoretical frameworks, reflecting the hybrid and evolving nature of PAT. For this reason, while alignment between theoretical models and evaluation strategies may enhance construct validity and facilitate the identification of mechanisms of change (12), such an alignment remains partial and inconsistently implemented in the current literature. Existing reviews have primarily focused on specific aspects of PAT assessment, including experiential measures from psychedelic research and clinical trials (13), therapeutic models (10), clinical efficacy (15), and the quality of reporting on psychological interventions (16). However, a systematic mapping of evaluation methods across all phases of this complex intervention, from preparation to acute administration and subsequent integration, is still missing from the literature.

To address the methodological fragmentation, this scoping review maps the epistemic and clinical assessment frameworks in the field, guided by two research questions: (1) What is the current state of the scientific literature on the methodologies and frameworks used to evaluate PAT across all phases of the complex intervention? (2) What are the main methodological gaps, and what priorities emerge for developing integrated, standardized assessment models?

The objectives of this review align with these questions and are aimed at bridging clinical and epistemic domains. Specifically, the objectives are to: (i) identify and characterize assessment instruments and frameworks across the preparatory, acute, and integration phases; (ii) describe clinical intervention models and competency frameworks; (iii) synthesize evidence on the measurement of clinical (standardized symptom and diagnostic measures), psychological (process and mechanistic constructs, such as psychological flexibility and connectedness), and existential outcomes; (iv) examine approaches to safety, ethics, and implementation evaluation; and (v) identify gaps in methodological integration and their implications for developing coherent, multidimensional evaluation models. In line with scoping review methodology, this study maps the extent of available evidence without formally appraising the clinical validity of the interventions.

## 2 Methods

This scoping review was conducted and reported in accordance with the PRISMA-ScR (Preferred Reporting Items for Systematic reviews and Meta-Analyses extension for Scoping Reviews) guidelines (17) and follows the Population, Concept, and Context (PCC) framework. The completed PRISMA-ScR checklist is provided in the **Appendix**. The Population included human participants of any age or clinical status, covering both clinical populations and healthy volunteers, engaged in PAT; the Concept encompassed methods, tools, and frameworks used for assessment and monitoring across all phases of the intervention process, including preparatory screening, acute experience, therapeutic processes, integration, clinical outcomes, and safety; and the Context was any clinical or research setting where PAT is administered and evaluated.

Peer-reviewed PAT publications published between 2008 and 2026 were eligible if they addressed assessment methods, psychometric instruments, clinical frameworks, or outcome measures in PAT. To ensure relevance, evaluation or assessment was required to constitute a central component of the publication’s aims or methodology. Foundational instrument-development and validation publications predating 2008 were additionally retained when necessary to document the provenance and psychometric basis of established measures identified in the PAT literature. The relevance of these foundational sources was additionally verified through backward citation checking of the included PAT publications. Instruments assessing nonordinary states of consciousness without an explicit link to a structured PAT intervention were excluded. Eligible study types included systematic reviews, meta-analyses, primary studies developing or validating assessment instruments used in PAT, methodological studies and critiques addressing evaluation or reporting practices, and clinical guidelines or frameworks with an explicit assessment component. Because the corpus included both primary studies and secondary evidence syntheses, publications were treated as the unit of analysis. Primary trials represented within systematic reviews or meta-analyses were therefore retained rather than de-duplicated at the study level, in accordance with the descriptive and mapping purposes of the scoping review. The original complex-intervention article and its corrigendum were treated as linked reports and were not interpreted as independent substantive evidence. PAT was defined as the administration of classic serotonergic psychedelic compounds or MDMA within a structured clinical context including preparatory, acute, and integrative phases (4, 5, 10), regardless of the degree of psychotherapeutic structuring. Studies were excluded if they focused on nontherapeutic use, purely pharmacological mechanisms without a clinical assessment component, or preclinical research, or were nonpeer-reviewed articles (e.g., editorials or conference abstracts without full text).

**Figure 1:**
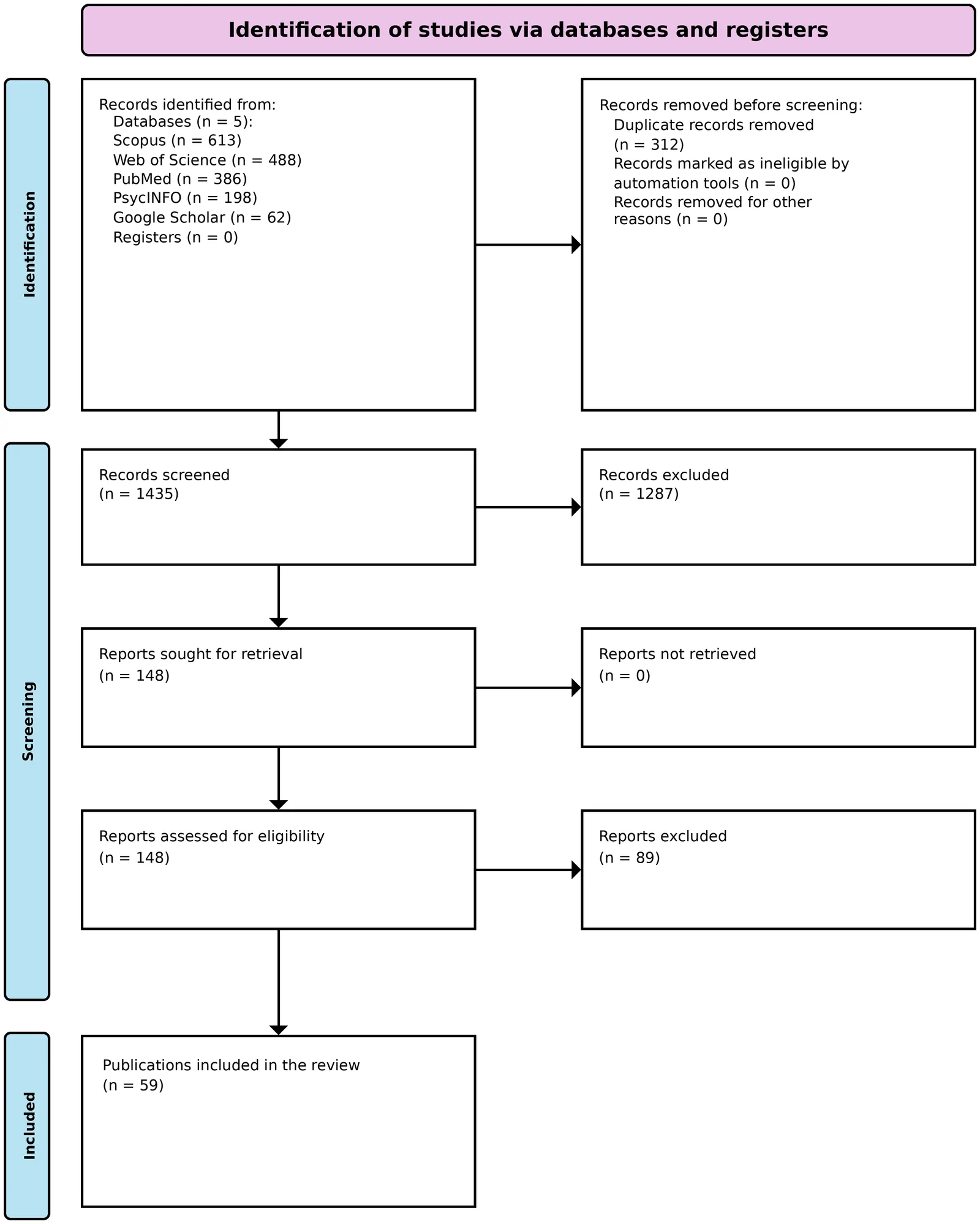
PRISMA flow diagram illustrating the study selection process, including identification, screening, eligibility assessment, and inclusion of the final 59 publications (64).

A comprehensive search was conducted in April 2026 across PubMed, Scopus, Web of Science, PsycINFO, and Google Scholar. Google Scholar was used as a supplementary source to identify potentially relevant literature not indexed in the primary databases. A core search strategy was developed using a Boolean expression with free-text keywords (**Table 1**), which was then adapted for each database using appropriate syntax and controlled vocabulary terms (e.g., Medical Subject Headings [MeSH]). A representative search string was: (“psychedelic-assisted therapy” OR “psychedelic therapy” OR psychedelics) AND (assessment OR evaluation OR measurement OR “process measures” OR “outcome measures” OR integration OR safety). The initial search yielded 1747 records. After removing 312 duplicates, two reviewers independently screened 1435 records by title and abstract, excluding 1287. Records were excluded at this stage primarily on the basis of nonrelevance to PAT as a structured intervention, a singular focus on purely pharmacological mechanisms without a psychotherapeutic component, preclinical design, nontherapeutic or recreational use contexts, a nonpeer-reviewed format, or an exclusive focus on compounds outside the defined scope of the review. The full texts of the remaining 148 articles were assessed for eligibility, with 89 being excluded. A final corpus of 59 publications was thus included in the synthesis (**Figure 1**).

**Table 1:** Search strategy.

Database	Search string	Date of search	Records retrieved
PubMed	(“psychedelic-assisted therapy” OR “psychedelic therapy” OR psychedelics) AND (assessment OR evaluation OR measurement OR “process measures” OR “outcome measures” OR integration OR safety)	April 2026	386
Scopus	TITLE-ABS-KEY(“psychedelic-assisted therapy” OR “psychedelic therapy” OR psychedelics) AND TITLE-ABS-KEY(assessment OR evaluation OR measurement OR “process measures” OR “outcome measures” OR integration OR safety)	April 2026	613
Web of Science	TS = (“psychedelic-assisted therapy” OR “psychedelic therapy” OR psychedelics) AND TS = (assessment OR evaluation OR measurement OR “process measures” OR “outcome measures” OR integration OR safety)	April 2026	488
PsycINFO	(“psychedelic-assisted therapy” OR “psychedelic therapy” OR psychedelics) AND (assessment OR evaluation OR measurement OR “process measures” OR “outcome measures” OR integration OR safety)	April 2026	198
Google Scholar	(“psychedelic-assisted therapy” OR “psychedelic therapy”) AND (assessment OR evaluation OR measurement OR “outcome measures” OR integration OR safety)	April 2026	62
Total			1,747

Data were charted by two reviewers (MLM and SF) who independently extracted information on study characteristics, assessment instruments, psychometric properties, populations studied, and relevant findings into a standardized data extraction form, with discrepancies being resolved through discussion. For each assessment instrument, we additionally charted the intervention phase addressed, the construct assessed, the provenance of the instrument (PAT-specific, psychedelic-specific and PAT-relevant, or borrowed or adapted from general psychiatry, psychology, psychotherapy, or medicine), the type of measure (self-report, clinician-rated, interview, or framework), and its psychometric and PAT-validation status. A narrative synthesis was conducted using a structured thematic approach. Findings were initially organized into four predefined domains derived deductively (top-down) from the review’s research questions and the sequential phases of the clinical process: (i) assessment instruments for subjective experiences; (ii) clinical intervention models and competency frameworks; (iii) clinical and existential outcome measures; and (iv) safety, ethics, and implementation frameworks. During synthesis, categories were iteratively refined to ensure alignment with the extracted data. Each publication was assigned to the domain that best represented its primary contribution to PAT assessment. Detailed charted characteristics of all 59 included publications, including study design, population, psychedelic compound, intervention phase, assessment instruments or frameworks, and principal findings, are provided in **Supplementary Table 1**.

In line with established scoping review methodology (17), no formal quality appraisal or risk-of-bias assessment of the included studies was conducted, as the primary goal was to map the extent, range, and nature of the available evidence rather than to evaluate the methodological quality of individual studies. No review protocol was prospectively registered.

## 3 Results

The findings from the 59 included publications are presented descriptively according to the predefined domains aligned with the review’s PCC framework. The complete study-level data charting is presented in **Supplementary Table 1**. The corpus comprised validation studies (n = 13), reviews and evidence syntheses (n = 20), meta-analyses and umbrella reviews (n = 3), methodological publications (n = 12), clinical guidelines and frameworks (n = 7), and primary studies (n = 4) (**Tables 2**, **3**). A notable increase in the number of publications was observed after 2019, addressing classic serotonergic psychedelic compounds (psilocybin, LSD, DMT) and MDMA across diverse populations. Assessment methods were applied across these substances within structured clinical settings. A phase-by-instrument assessment matrix mapping these instruments across the intervention phases, together with their provenance and validation status within PAT, is presented in **Table 4**.

**Table 2:** Characteristics of the included publications.

Category	Number of publications (n = 59)	Description	Publications included in each category
Validation Studies	13	Development and validation of psychedelic-specific, clinical, relational, preparatory, and existential measures; cross-cultural adaptations	(18–30)
Reviews and Evidence Syntheses	20	Reviews of clinical outcomes, spirituality, trauma, adverse events, demographic gaps	(2, 8, 11, 13–15, 31–44)
Meta-Analyses and Umbrella Reviews	3	Quantitative syntheses of clinical efficacy, anxiety outcomes, and methodological factors	(1, 45, 46)
Methodological Publications	12	Analyses of reporting quality, clinical intervention fidelity, replication expectations, and study design	(3, 7, 9, 12, 16, 47–53)
Clinical Guidelines and Frameworks	7	Regulatory, ethical, competency-based, and implementation frameworks	(10, 54–59)
Primary Studies	4	Primary clinical trials and empirical studies charted for their assessment approaches	(5, 60–62)

Conceptual and methodological publications addressing reporting quality, intervention fidelity, study protocols, replication expectations, trial design, and related corrections.

**Table 3:** Synthesis of findings by evaluation domain.

Domain	Number of publications (n = 59)	Key instruments and frameworks	Main findings	Gaps identified
1. Assessment instruments for subjective experiences	18	MEQ-30 (30), CEQ (28), EBI (27), PIS (25), PPS (24), IPPS (18)	Cross-cultural validation of instruments for mystical and challenging experiences; standardized characterization of the acute psychedelic state	Limited validation in non-Western and clinical populations; limited coverage of the preparatory and integration phases
2. Clinical intervention and associated competency frameworks	13	EMBARK (10), THRIVE (54), WAI (23), competency framework (58)	Preliminary identification of core practitioner competencies; articulation of the “therapeutic container” as a structural element	Absence of PAT-specific process and fidelity measures
3. Clinical and existential outcome measures	21	BDI (19), HAM-D (22), CAPS (20), FACIT-Sp (26), WCS (29), Psychedelic Integration Scales (IES/EIS; 21)	Reported symptom reductions across depression, anxiety, PTSD, and existential distress; spiritual and existential outcomes acknowledged	Inconsistent use of existential and spiritual measures; underrepresentation of post-session integration outcomes; demographic narrowness of the research base
4. Safety, ethics, and implementation frameworks	7	ReSPCT checklist (50); regulatory taxonomies (56); clinical practice guidelines (59); digital implementation models (35)	Ethical and regulatory frameworks articulated; emerging implementation models including digitally enabled delivery	Variability in adverse event monitoring and reporting; implementation science in early stages; limited empirical validation of ethical frameworks

PAT, psychedelic-assisted therapy; MEQ-30, 30-item Mystical Experience Questionnaire; CEQ, Challenging Experience Questionnaire; WAI, Working Alliance Inventory; EBI, Emotional Breakthrough Inventory; PIS, Psychological Insight Scale; PPS, Psychedelic Preparedness Scale; IPPS, Imperial Psychedelic Predictor Scale; BDI, Beck Depression Inventory; HAM-D, Hamilton Depression Rating Scale; CAPS, Clinician-Administered PTSD Scale; FACIT-Sp, Functional Assessment of Chronic Illness Therapy–Spiritual Well-Being Scale; WCS, Watts Connectedness Scale; IES, Integration Engagement Scale; EIS, Experienced Integration Scale; PTSD, post-traumatic stress disorder.

**Table 4:** Phase-by-instrument assessment matrix.

Instrument (ref.)	Construct assessed	Provenance	Measure type	Validation status within PAT
Screening and preparation (pre-session)				
Structured clinical and diagnostic interviews (1)	Psychiatric eligibility; baseline symptomatology	Borrowed (psychiatry)	Clinician-administered interview	Standardized in general use; not PAT-specific
Medical screening: physical examination, laboratory tests, cardiovascular monitoring (51)	Physiological safety and eligibility	Borrowed (medicine)	Clinical procedures	Standardized; not PAT-specific
PPS (24)	Readiness for the psychedelic session	Psychedelic-specific and PAT-relevant	Self-report	Developed and psychometrically validated for the preparatory phase
IPPS (18)	Predicted response (set, rapport, intention)	Psychedelic-specific and PAT-relevant	Self-report (prospective)	9-item, three-factor scale; validated across four datasets (naturalistic and controlled administration)
Expectancy-assessment tools (9, 18)	Treatment expectations	Borrowed and adapted	Self-report and qualitative	Exploratory; limited PAT validation
Acute experience				
MEQ (30)	Mystical-type experience (30 items, four factors)	Psychedelic-specific and PAT-relevant	Self-report	Validated (MEQ-30); Japanese cross-cultural adaptation
CEQ (28)	Challenging/difficult experience (seven domains)	Psychedelic-specific and PAT-relevant	Self-report	Validated; Japanese cross-cultural adaptation
EBI (27)	Emotional release during the acute experience	Psychedelic-specific and PAT-relevant	Self-report	Validated; predictive of post-experience well-being
Therapeutic process				
WAI (23)	Therapeutic alliance and therapeutic relationship	Borrowed (general psychotherapy)	Self-report	Validated in general psychotherapy; adapted, not PAT-validated
n/a	No validated PAT-specific process measure identified (12, 34)	n/a	n/a	Gap
Integration (post-session)				
PIS (25)	Psychological insight following the experience	Psychedelic-specific and PAT-relevant	Self-report	Validated
WCS (29)	Connectedness to self, others, and world	Psychedelic-specific and PAT-relevant	Self-report	Validated, including a dataset from a randomized controlled trial comparing psilocybin with escitalopram
IES/EIS (21)	Integration behaviors (IES) and experienced integration (EIS)	Psychedelic-specific and PAT-relevant	Self-report	Two scales; validated via exploratory and confirmatory factor analysis and convergent validity
Clinical and existential outcomes				
BDI (19)	Depression severity	Borrowed (psychiatry)	Self-report	Validated in general use; not PAT-specific
HAM-D (22)	Depression severity	Borrowed (psychiatry)	Clinician-rated	Validated in general use; not PAT-specific
CAPS (20)	PTSD symptoms	Borrowed (psychiatry)	Clinician-administered interview	Validated in general use; not PAT-specific
FACIT-Sp (26)	Spiritual well-being, including meaning, peace, and faith	Borrowed from oncology and palliative care	Self-report	Validated in general use; not PAT-specific
Existential/spiritual and personal-meaning measures (36)	Spiritual well-being	Borrowed and adapted from oncology and palliative care	Self-report	Validated in general use; adapted for PAT
Safety, ethics, and implementation				
General adverse-event checklists with open-ended questions (51)	Adverse events	Borrowed (medicine)	Checklist and qualitative	No validated PAT-specific adverse-event instrument available
Risk–benefit assessments (32); seizure-risk reviews (42)	Risk profile	Framework and review	Narrative	Not a psychometric instrument
Ethical frameworks and clinical practice guidelines (55, 59); regulatory taxonomies (56)	Ethical and regulatory governance	Framework	Conceptual	No validated effectiveness measure
Informed-consent quality measures (55)	Quality of the informed-consent process	n/a	n/a	Validated measures limited or absent
Implementation frameworks; digitally enabled at-home models (35); consensus statement (57)	Implementation models	Framework	Conceptual	Emerging

Instruments and frameworks identified across the intervention phases, with their provenance (PAT-specific, psychedelic-specific and PAT-relevant, or borrowed or adapted from general psychiatry, psychology, psychotherapy, or medicine) and validation status within psychedelic-assisted therapy (PAT).

PAT, psychedelic-assisted therapy; PPS, Psychedelic Preparedness Scale; IPPS, Imperial Psychedelic Predictor Scale; MEQ, Mystical Experience Questionnaire (MEQ-30, 30-item version); CEQ, Challenging Experience Questionnaire; EBI, Emotional Breakthrough Inventory; WAI, Working Alliance Inventory; PIS, Psychological Insight Scale; WCS, Watts Connectedness Scale; IES, Integration Engagement Scale; EIS, Experienced Integration Scale; BDI, Beck Depression Inventory; HAM-D, Hamilton Depression Rating Scale; CAPS, Clinician-Administered PTSD Scale; FACIT-Sp, Functional Assessment of Chronic Illness Therapy–Spiritual Well-Being Scale; PTSD, post-traumatic stress disorder.

### 3.1 Assessment instruments for subjective experiences and acute phenomena

This domain is characterized by a small number of validated, psychedelic-specific questionnaires widely used in PAT research. The most prominent is the Mystical Experience Questionnaire (MEQ; 30), a 30-item, four-factor instrument designed to measure the intensity of mystical-type experiences, which have frequently been associated with therapeutic outcomes. Recent work has focused on its cross-cultural adaptation and validation, including in Japanese populations (30).

Complementing the MEQ, the Challenging Experience Questionnaire (CEQ; 28) is a psychedelic-specific instrument widely used in PAT research to assess difficult or distressing aspects of the psychedelic experience. The CEQ quantifies seven domains of challenging experiences, including fear, grief, and paranoia, and has undergone cross-cultural validation in Japanese populations (28). Together, the MEQ and CEQ characterize distinct and potentially overlapping dimensions of the acute psychedelic experience. CEQ-assessed challenging experiences should not be interpreted solely as adverse outcomes, because their clinical significance may depend on the therapeutic support provided during the session and their subsequent integration (14, 28). A recent review of patient-reported outcome measures for the acute subjective experience confirmed the dominance of these instruments in contemporary clinical research (14).

Beyond these core instruments, the literature describes other measures. An integrative review by Baker et al. (31) mapped the variety of instruments used to assess spiritual dimensions in patients with serious illness, highlighting the use of both validated and adapted measures. These include general measures of spirituality, such as the Functional Assessment of Chronic Illness Therapy Spiritual Well-Being Scale (FACIT-Sp; 26), which have been adapted for use in PAT studies. Compared with the mature measurement of acute experiences, fewer instruments have been validated for the preparatory and integration phases, and the available scales have not yet been widely replicated across clinical PAT contexts (21, 24, 25, 29). Many studies in these areas continue to rely on exploratory or adapted measures, including qualitative interviews and general measures of psychological well-being (35), indicating that these phases remain methodologically less developed.

The pre-session screening phase is addressed in the literature through a combination of standardized psychiatric diagnostic tools and medical screening procedures. Studies consistently report the use of structured clinical interviews, such as standardized diagnostic interviews, to establish psychiatric eligibility and assess baseline symptomatology (1). Medical screening typically includes physical examinations, laboratory tests, and cardiovascular monitoring to ensure participant safety prior to substance administration (51). Expectancy assessment, when reported, was conducted through self-report questionnaires or qualitative inquiry, although the assessment of expectancy remains methodologically heterogeneous in PAT research (9, 18). The methodological status of pre-session instruments ranges from standardized to exploratory measures. While psychiatric diagnostic scales and medical procedures tend to be standardized and widely accepted (1), tools for assessing psychological preparedness and treatment expectations remain exploratory and present limited psychometric validation within PAT research (34).

### 3.2 Clinical intervention models and competency frameworks

Several studies (10, 54, 58, 60) addressed the therapeutic framework, indicating that this is a developing yet methodologically exploratory domain. The therapeutic setting was consistently described as being fundamental to PAT. The literature contains several proposed therapeutic models that provide a conceptual framework for the clinical intervention process. One of the most detailed is EMBARK (Existential–Spiritual, Mindfulness, Body-Aware, Affective–Cognitive, Relational, Keeping Momentum), a transdiagnostic and trans-drug model that outlines a broad approach to PAT, including elements of psychoeducation and somatic awareness (10). Another is THRIVE (The Outdoors, Holistic Health, Relationships, Internal Self, Values, Existential Meaning) (54), which is aimed at bridging indigenous knowledge with evidence-based practice, emphasizing a culturally sensitive approach to psychedelic integration. These frameworks all emphasize the importance of the “therapeutic container,” the structured relational safety within which the psychedelic experience unfolds (10). From an assessment perspective, the therapeutic container may therefore encompass the quality of the therapeutic alliance, perceived relational and environmental safety, therapist adherence, and continuity of support across the preparation, dosing, and integration phases (10, 23, 50).

The corpus further includes instruments relevant to PAT spanning several intervention phases. Emotional release during the acute experience is quantified by the Emotional Breakthrough Inventory [EBI; (27)], and psychological insight arising during the session by the Psychological Insight Scale [PIS; (25)]. For the pre-session phase, the Psychedelic Preparedness Scale [PPS; (24)] assesses readiness, and the Imperial Psychedelic Predictor Scale [IPPS; (18)] provides a prospective estimate of response. For post-session integration, the Watts Connectedness Scale [WCS; (29)] measures a felt sense of connectedness to self, others, and the wider world, and the Psychedelic Integration Scales (21) assess integration behaviors and experiences. The systematic appraisal of these instruments within integrated PAT evaluation frameworks remains a priority for future work (14).

Alongside the reviewed conceptual models, preliminary convergence was suggested around core competencies for PAT practitioners, although this observation rests largely on a single study and should be interpreted with caution (58). These competencies, which have not as yet been formalized into a standardized assessment tool, include a combination of general clinical intervention skills and specific knowledge related to psychedelic work, such as the ability to manage challenging experiences and facilitate integration. Empirical studies have documented provider perspectives on treatment challenges (60) and the need for specialized training (58), reinforcing the importance of establishing a standardized competency framework. The literature also includes quantitative evidence examining the relationship between clinical intervention duration and outcomes. A recent meta-analysis found no statistically significant association between the number of therapy hours and treatment outcomes, while highlighting inadequate reporting and a lack of standardized therapy protocols (46).

Despite the conceptual focus on the clinical intervention process, the development of validated, PAT-specific instruments for measuring process variables is still an emerging area. Most studies adapt measures from general psychotherapy research, such as the Working Alliance Inventory [WAI; (23)], to assess the clinical relationship in PAT. Although these adapted measures provide data on general relational factors, their capacity to capture the specific dynamics of the clinical intervention in PAT (12, 34) remains limited. Critiques of the rigor in assessing clinical interventions (12, 34) and trial design (3) highlight the need for stronger and PAT-specific process measures. The evaluation of the clinical intervention process is characterized by a divergence between its conceptual frameworks and its current methodological development.

### 3.3 Clinical and existential outcome measures

The corpus includes systematic reviews and meta-analyses examining clinical outcomes in PAT (1). Studies within this domain commonly use standardized and validated instruments as primary measures of psychiatric symptoms. These include the Beck Depression Inventory [BDI; (19)], the Hamilton Depression Rating Scale [HAM-D; (22)], and the Clinician-Administered PTSD Scale [CAPS; (20)]. Reviews have reported symptom reductions in depression and anxiety (15), trauma-related disorders (37), and anxiety, depression, and existential distress among people with life-threatening illnesses (2). Some meta-analyses have reported large between-group effect sizes; however, comparisons with standard psychopharmacological treatments remain methodologically constrained (1, 45). An umbrella review synthesizing 23 meta-analyses of randomized controlled trials, using the Grading of Recommendations Assessment, Development and Evaluation (GRADE) approach, provides a cross-indication assessment of psychedelic efficacy and safety across psychiatric conditions (45).

The literature describes a broader range of standardized clinical instruments used across PAT trials. These include both self-report and clinician-administered measures, reflecting a multi-perspective approach to outcome assessment. Commonly used instruments include dedicated clinician-administered measures of post-traumatic stress disorder symptoms (1). The selection of instruments across studies indicates a reliance on established psychiatric outcome measures, utilized within structured clinical protocols.

The literature also describes the potential impact of PAT on existential outcomes, with systematic reviews reporting improvements in quality of life and mystical experiences (36). The measurement of these constructs is described as less standardized than for clinical symptoms, and a mix of validated and exploratory instruments is used, including measures of spiritual well-being and personal meaning. The application of these measures is inconsistent across studies, which hinders cross-study comparisons and meta-analytic integration (36), highlighting a need for the development and validation of PAT-specific measures of existential outcomes.

A limitation identified across the outcome literature is the limited demographic diversity in clinical trials. One review noted a severe underrepresentation of older adults (33), and some other studies (e.g., 1, 11) have highlighted the limited racial and ethnic diversity among trial participants. This limited representation constrains the generalizability of findings and raises questions about the applicability of PAT to a wider range of populations. The field has begun to acknowledge this limitation, with increasing calls for more inclusive research practices (33).

### 3.4 Safety monitoring, adverse event assessment, and implementation frameworks

A smaller subset of studies (e.g., 35, 42, 56) focused on safety and implementation, highlighting this as a methodologically less developed area of inquiry. The literature includes studies addressing safety and implementation, focusing on the systematic monitoring of adverse events and the development of implementation frameworks. The domain is characterized by calls for standardized protocols for documenting and grading physiological and psychological adverse events. The literature indicates that a standardized, PAT-specific instrument for assessing adverse events is still missing from the literature (51). A systematic review of psilocybin-assisted psychotherapy further documents variability in the definition and measurement of adverse events across trials, indicating the need for standardized safety reporting frameworks (38), while a cross-sectional analysis of ClinicalTrials.gov registrations identified systematic inconsistencies between registered protocols and published safety and outcome data in MDMA and psilocybin trials (52). Consequently, studies typically use a combination of general medical adverse event checklists and open-ended questions, which impacts the systematic analysis and comparison of safety data across trials (51). The literature also includes risk–benefit assessments (32) and reviews of specific risks such as seizure potential (42).

Ethical considerations are a prominent theme in this domain (55), leading to the development of clinical practice guidelines (58) and regulatory taxonomies to inform policy (56). These conceptual frameworks provide a basis for the ethical implementation of PAT, while the development of empirical tools for measuring their effectiveness remains an ongoing process (55). For example, while guidelines emphasize the importance of informed consent, the availability of validated measures for assessing the quality of the informed consent process in PAT remains limited (55).

An emerging area is the assessment of implementation models that diverge from traditional in-clinic settings, such as digitally enabled at-home therapy (35). A multidisciplinary consensus statement from a national network of depression centers further identifies the gaps in implementation frameworks required for the responsible integration of psilocybin into routine clinical care (57). Such decentralized implementation models raise new safety and ethical challenges that require the development of novel evaluation methods. Critiques of trial reporting practices (34) and guidance for replication studies (47) further highlight the need for increased methodological rigor in this domain. Overall, the assessment of the safety and implementation domain is characterized by a divergence between its conceptual and ethical frameworks and its current empirical and methodological development, a gap increased by the absence of a unified causal model (3, 10, 12).

## 4 Discussion

This scoping review maps a scientific field actively constructing the methodological foundations for PAT research and practice, highlighting the current absence of a unified causal model. The analysis of 59 publications reveals progression from an initial focus on establishing clinical efficacy to an inquiry into the processes and implementation challenges of this clinical intervention. The findings directly address the two guiding research questions of this review.

### 4.1 Current state of the literature on evaluation methodologies and frameworks

In response to the first research question regarding the current state of the scientific literature on the methodologies and frameworks used to evaluate PAT across all phases of the intervention, the literature indicates developments in specific domains. The development and cross-cultural validation of instruments for quantifying subjective phenomena, such as the MEQ (30) and the CEQ (28), contribute to the development of a standardized language for characterizing the acute psychedelic state. Concurrently, clinical researchers have developed therapeutic frameworks that emphasize the role of the “therapeutic container” (10) and have begun to define practitioner competencies (58). More recently, an international Delphi consensus produced the Reporting of Setting in Psychedelic Clinical Trials (ReSPCT) checklist, a set of 30 extra-pharmacological variables recommended for standardized reporting of settings in psychedelic clinical trials (50). Evidence from meta-analyses has addressed the clinical efficacy of PAT for several psychiatric conditions (1), utilizing established clinical symptom measures.

### 4.2 Methodological gaps and priorities for integrated assessment models

With regard to the second research question concerning methodological gaps and priorities, the analysis indicates limited integration across assessment domains. A primary observation is the absence of integrated evaluation protocols that connect measurement across different intervention phases. Pre-session screening, acute experience measurement, process monitoring, and outcome assessment are typically conducted as separate activities. Another area for development is the validation of tools for measuring the process and outcomes of post-session integration, a phase considered important for long-term clinical outcomes (10). Although the role of existential and spiritual outcomes is acknowledged, the measures used are employed less consistently than clinical symptom scales (36). The assessment of the clinical process itself relies on measures adapted from general clinical intervention research, indicating a potential role for instruments designed for and validated within PAT contexts. Furthermore, the methods for monitoring and reporting adverse events vary across studies (51), which impacts the understanding of safety profiles. The development of the ReSPCT checklist (50) represents a step toward addressing this gap, though its adoption across research groups is yet to be established. Finally, the development of implementation science and service assessment remains in its early stages (35).

Addressing these gaps necessitates moving from parallel, fragmented assessment methods to an integrated, multidimensional assessment model, which should connect data across all intervention phases and evaluation domains. Achieving this will require the continued validation of existing instruments in diverse and underrepresented populations, as the research base remains demographically narrow (33). It also calls for the development of novel, validated instruments for the undermeasured domains of post-session integration and existential outcomes, the creation of PAT-specific intervention process measures, and the standardization of safety monitoring protocols. For researchers, this review highlights the need to design trials that incorporate multi-domain assessment protocols; for clinicians, it points to the available validated instruments while indicating the need for increased attention to process and safety monitoring; and for regulatory and policy stakeholders, it provides an evidence-based rationale for developing clear clinical practice guidelines and well-defined implementation frameworks to guide the responsible translation of PAT into mainstream healthcare.

A central issue underlying the observed methodological fragmentation is the absence of a shared causal model of therapeutic change in PAT. Multiple mechanisms have been proposed in the literature, including pharmacologically induced modulations in consciousness, emotional breakthrough, psychological flexibility, expectancy effects, and relational processes. However, these elements are often investigated in isolation and without a unifying theoretical framework, and as a result, current evaluation strategies tend to reflect domain-specific priorities, leaving aside an integrated account of how therapeutic outcomes emerge. This lack of a coherent causal architecture limits the interpretability of findings and complicates the comparison and synthesis of results across studies.

The development of integrated assessment models therefore depends on building theoretically grounded causal pathways that connect the acute experience to long-term clinical outcomes. In the absence of such models, measurement remains descriptive rather than explanatory, and the relationship between process variables and outcome measures cannot be fully specified. Future research should prioritize the construction and empirical testing of multidimensional causal frameworks capable of capturing the dynamic interaction between pharmacological action, therapeutic context, and individual psychological processes. Such an approach would support the development of shared evaluation criteria and strengthen the empirical basis of the field.

### 4.3 Mechanisms of therapeutic change

The evaluation of PAT requires a systematic understanding of the causal mechanisms underlying therapeutic outcomes. Muthukumaraswamy and colleagues (3) describe PAT as a complex intervention in which compound-induced activation, psychological processing, and contextual modulation operate as interacting components. Compound-induced activation elicits nonordinary states of consciousness, documented through the measurement of mystical experiences (30, 36) and emotional breakthroughs (27). These acute subjective phenomena are theorized to function as catalysts for psychological restructuring (4, 10, 37), and it has also been proposed that the relationship between acute psychedelic-induced effects and sustained clinical outcomes involves psychological processing as an intervening pathway (3, 10). Variables such as psychological flexibility and pre-session expectancy effects are proposed as modulators of an individual’s capacity to integrate the compound-induced experience into coherent cognitive and emotional frameworks (3, 10, 18). Contextual modulation is conceptualized as the structural container for this process: The therapeutic alliance has been described as a relational matrix associated with the regulation of the acute experience and the facilitation of post-session integration (10, 23). Consequently, multidimensional evaluation models are called upon to address the interaction among these variables, treating compound-induced effects and psychological processes as components of an integrated causal structure, albeit as yet incompletely specified (12, 34).

### 4.4 Limitations

This scoping review has several limitations. The search may have missed relevant gray literature, and no formal quality appraisal of the included studies was performed, in line with scoping review methodology. The heterogeneity of the studies prevented quantitative meta-analysis, and the rapid pace of research in the field means that new studies may have been published after the search period had concluded. Furthermore, because the corpus includes both primary studies and secondary syntheses (systematic reviews and meta-analyses), some primary trials may be represented more than once across the evidence base; these overlapping sources were not de-duplicated, consistent with the descriptive aim of a scoping review. A further limitation concerns the exclusion of ketamine-based interventions. Although ketamine has generally been investigated within a different therapeutic framework, distinguishing ketamine-assisted psychotherapy from conventional pharmacological ketamine treatment would have required substantial additional work; its exclusion therefore narrows the scope of the present review (63).

## 5 Conclusions

This scoping review maps the current state of assessment methodology in psychedelic-assisted therapy, understood as a complex intervention integrating compound-induced, psychological, and contextual components. The literature documents advances in the development and validation of instruments for characterizing acute subjective states, articulating clinical intervention models, and establishing outcome data across several psychiatric conditions. The methodological picture presents areas of unresolved fragmentation: Assessment domains across intervention phases remain largely disconnected, measures for post-session integration and existential outcomes are employed inconsistently, and safety monitoring protocols vary across studies. Demographic representation in the research base is geographically and socioculturally narrow. Progress toward integrated, multidimensional assessment models that are capable of capturing the interaction among compound-induced effects, relational processes, and cognitive mediators represents a methodological priority identified across the reviewed literature. This review provides a structured account of these priorities to support the development of evaluation frameworks that are adequate given the epistemological complexity of PAT, and the absence of a unified causal model linking compound-induced effects, psychological processes, and clinical outcomes remains a central challenge for the development of integrated evaluation frameworks.

## Data Availability

The original contributions presented in the study are included in the article/**Supplementary Material**. Further inquiries can be directed to the corresponding author.

## Appendix

**Table A1:** PRISMA-ScR Checklist (17).

Section/Item	Reported in
1. Title: identifies the report as a scoping review	Title
2. Structured summary	Abstract
3. Rationale	Introduction
4. Objectives: review questions and objectives (population/concept/context)	Introduction (two research questions; objectives i–v; PCC framework)
5. Protocol and registration	Methods (no protocol was prospectively registered)
6. Eligibility criteria	Methods (inclusion and exclusion criteria)
7. Information sources	Methods; **Table 1** (five databases; last search April 2026)
8. Search: full search strategy for at least one database	**Table 1**
9. Selection of sources of evidence	Methods; **Figure 1**
10. Data charting process	Methods (two independent reviewers; standardized charting form)
11. Data items	Methods (intervention phase, construct, provenance, measure type, psychometric and PAT-validation status)
12. Critical appraisal of individual sources of evidence (optional)	Methods and Limitations (no formal appraisal, consistent with scoping review methodology)
13. Synthesis of results: methods of handling and synthesizing data	Methods (narrative synthesis; four evaluation domains)
14. Selection of sources of evidence: numbers screened, assessed, included	Results; **Figure 1** (1,747 identified; 89 excluded at full text; 59 included)
15. Characteristics of sources of evidence	**Supplementary Table 1**, **Table 2**
16. Critical appraisal within sources of evidence (optional)	Not applicable
17. Results of individual sources of evidence	**Supplementary Table 1**
18. Synthesis of results	Results; **Tables 3**, **4**
19. Summary of evidence	Discussion
20. Limitations	Limitations
21. Conclusions	Conclusions
22. Funding	This research received no external funding

PAT, psychedelic-assisted therapy; PCC, Population, Concept, and Context.
