# Supplementary material for: Methodological fragmentation and integration priorities in psychedelic-assisted therapy assessment: a scoping review
Source: Front Psychiatry. 2026 Aug 6;17:1880744. doi: 10.3389/fpsyt.2026.1880744 (PMC13490194; doi:10.3389/fpsyt.2026.1880744)
Supplement: Supplementary file 1 [file Table1.docx]

**Supplementary Table S1. Detailed charted characteristics of the included publications**

Note. Reference numbers correspond to the bibliography of the manuscript. BDI = Beck Depression Inventory; CAPS = Clinician-Administered PTSD Scale; CEQ = Challenging Experience Questionnaire; EBI = Emotional Breakthrough Inventory; EIS = Experienced Integration Scale; EMBARK = Existential–Spiritual, Mindfulness, Body-Aware, Affective–Cognitive, Relational, Keeping Momentum; FACIT-Sp = Functional Assessment of Chronic Illness Therapy–Spiritual Well-Being; GRADE = Grading of Recommendations Assessment, Development and Evaluation; HAM-D = Hamilton Depression Rating Scale; IES = Integration Engagement Scale; IPPS = Imperial Psychedelic Predictor Scale; MDMA = 3,4-methylenedioxymethamphetamine; MEQ = Mystical Experience Questionnaire (MEQ-30 = 30-item version); PAT = psychedelic-assisted therapy; PAP = psychedelic-assisted psychotherapy; PIS = Psychological Insight Scale; PPS = Psychedelic Preparedness Scale; PTSD = post-traumatic stress disorder; ReSPCT = Reporting of Setting in Psychedelic Clinical Trials; THRIVE = The Outdoors, Holistic Health, Relationships, Internal Self, Values, Existential Meaning; WAI = Working Alliance Inventory; WCS = Watts Connectedness Scale.

| **Ref.** | **Publication** | **Publication type** | **Population/context and compound(s)** | **PAT phase(s) / evaluation domain** | **Instrument(s) / framework(s)** | **Main charted contribution / validation status** |
| --- | --- | --- | --- | --- | --- | --- |
| 1 | Luoma et al. (2020) | Meta-analysis | Placebo-controlled PAT trials; psychiatric populations; multiple psychedelic compounds | Clinical outcomes; safety | Standardized symptom and outcome measures across included trials | Pooled between-group effects and methodological constraints in placebo-controlled PAT trials. |
| 2 | Schipper et al. (2024) | Systematic review (Cochrane) | People with life-threatening diseases; psychedelic-assisted interventions | Clinical and existential outcomes; safety | Depression, anxiety, existential-distress, and safety outcomes | Synthesized evidence for anxiety, depression, and existential distress in serious medical illness. |
| 3 | Muthukumaraswamy et al. (2025) | Conceptual/methodological article | PAT trials across compounds and indications | All phases; causal model; trial design | Complex-intervention framework | Conceptualized PAT as an interaction among pharmacological action, psychological processing, and context; outlined trial-design implications. |
| 5 | Mitchell et al. (2021) | Phase 3 randomized controlled trial | Adults with severe PTSD; MDMA-assisted therapy | Preparation, dosing, integration; clinical outcomes; safety | CAPS and protocol-based clinical and safety assessments | Provided a structured MDMA-assisted therapy model and clinical outcome data for severe PTSD. |
| 7 | Zamaria et al. (2025) | Conceptual article | Psychedelic-assisted interventions across modalities | Therapeutic process; conceptual scope | PAT/PAP conceptual distinction; role of psychotherapy | Clarified the contribution of psychotherapy and the continuum of therapeutic structuring in psychedelic interventions. |
| 8 | Barstowe & Kajonius (2026) | Systematic review | Placebo-controlled psychedelic studies; multiple compounds | Trial design; masking/blinding | Masking and placebo-control procedures | Mapped masking practices and identified limitations in maintaining blinding. |
| 9 | Aday et al. (2022) | Methodological recommendations | Psychedelic clinical trials; multiple indications and compounds | Trial design; expectancy; masking | Methodological rigor framework | Proposed strategies to improve masking, expectancy control, measurement, and interpretability. |
| 10 | Brennan & Belser (2022) | Review and clinical framework | PAT across diagnoses and psychedelic compounds | Preparation, dosing, integration; therapeutic process | EMBARK model | Reviewed therapeutic models and introduced a transdiagnostic, trans-drug clinical framework. |
| 11 | Andersen et al. (2021) | Systematic review | Modern-era clinical studies of classic serotonergic psychedelics | Clinical outcomes; mechanisms; safety | Standardized clinical outcome measures across trials | Synthesized therapeutic effects and methodological characteristics of modern clinical studies. |
| 12 | Fradet (2025) | Methodological critique | Psychotherapy interventions embedded in psychedelic treatments | Therapeutic process; fidelity; reporting | Psychotherapy evaluation and reporting criteria | Identified weaknesses in evaluating and reporting psychological interventions and called for stronger process and fidelity measures. |
| 13 | Herrmann et al. (2023) | Scoping review | Psychedelic research and clinical trials; multiple compounds | Acute subjective experience | Experiential measures used in psychedelic research | Mapped available subjective-experience instruments and their use across research contexts. |
| 14 | Hovmand et al. (2024) | Review of patient-reported outcome measures | Clinical research on classical psychedelics | Acute subjective experience | MEQ, CEQ, and other acute-experience measures | Evaluated the coverage and measurement properties of instruments for acute psychedelic experiences. |
| 15 | Muttoni et al. (2019) | Systematic review | People treated for depression and anxiety; classic psychedelics | Clinical outcomes | Depression and anxiety outcome measures | Reviewed clinical evidence for classic psychedelics in depression and anxiety. |
| 16 | Seybert et al. (2025) | Systematic review | Psychedelic treatment studies with psychological interventions | Therapeutic process; fidelity; reporting | Reporting criteria for psychological interventions | Assessed completeness and quality of reporting of psychological support and psychotherapy components. |
| 18 | Angyus et al. (2024) | Instrument-validation study | Naturalistic and controlled psychedelic-administration datasets; multiple compounds | Screening and preparation | Imperial Psychedelic Predictor Scale (IPPS) | Validated a 9-item, three-factor prospective measure of set, rapport, and intention. |
| 19 | Beck et al. (1961) | Foundational instrument-development study | People with depressive symptoms; non-PAT source | Clinical outcomes | Beck Depression Inventory (BDI) | Established a self-report measure of depression severity later used in PAT research. |
| 20 | Blake et al. (1995) | Foundational instrument-development study | People with PTSD; non-PAT source | Clinical outcomes | Clinician-Administered PTSD Scale (CAPS) | Established a clinician-administered measure of PTSD symptoms later used in PAT trials. |
| 21 | Frymann et al. (2022) | Instrument-development and validation study | People reporting psychedelic experiences; multiple compounds | Post-session integration | Integration Engagement Scale (IES); Experienced Integration Scale (EIS) | Developed and validated complementary measures of integration behaviors and experienced integration. |
| 22 | Hamilton (1960) | Foundational instrument-development study | People with depression; non-PAT source | Clinical outcomes | Hamilton Depression Rating Scale (HAM-D) | Established a clinician-rated depression measure later used in PAT studies. |
| 23 | Horvath & Greenberg (1989) | Foundational instrument-validation study | General psychotherapy context; non-PAT source | Therapeutic process | Working Alliance Inventory (WAI) | Established a measure of therapeutic alliance subsequently adapted in PAT research. |
| 24 | McAlpine et al. (2024) | Instrument-development and validation study | People preparing for psychedelic experiences; psychedelic research context | Screening and preparation | Psychedelic Preparedness Scale (PPS) | Developed and psychometrically validated a measure of readiness for psychedelic sessions. |
| 25 | Peill et al. (2022) | Instrument-development and validation study | Psychedelic-experience datasets; multiple compounds | Acute experience and integration | Psychological Insight Scale (PIS) | Developed and validated a measure of psychological insight following psychedelic experiences. |
| 26 | Peterman et al. (2002) | Foundational instrument-validation study | People with cancer; non-PAT source | Existential and spiritual outcomes | FACIT-Sp | Validated a measure of spiritual well-being, meaning, peace, and faith later adapted in PAT studies. |
| 27 | Roseman et al. (2019) | Instrument-development and validation study | Psychedelic-experience participants; multiple compounds | Acute subjective experience | Emotional Breakthrough Inventory (EBI) | Validated a measure of emotional breakthrough and examined associations with later well-being. |
| 28 | Tani et al. (2024) | Cross-cultural instrument-validation study | Japanese-language psychedelic research sample | Acute subjective experience | Japanese Challenging Experience Questionnaire (CEQ) | Developed and validated a Japanese version of the CEQ. |
| 29 | Watts et al. (2022) | Instrument-development and validation study | Psychedelic-experience datasets, including a psilocybin-versus-escitalopram trial | Post-session integration; psychological outcomes | Watts Connectedness Scale (WCS) | Validated a measure of connectedness to self, others, and the world. |
| 30 | Yonezawa et al. (2024) | Cross-cultural instrument-validation study | Japanese-language psychedelic research sample | Acute subjective experience | Japanese 30-item Mystical Experience Questionnaire (MEQ-30) | Developed and validated a Japanese version of the MEQ-30. |
| 31 | Baker et al. (2023) | Integrative review | Psilocybin studies in people with serious illness | Existential and spiritual outcomes | Spirituality and spiritual-well-being measures | Mapped validated and adapted measures of spirituality used in serious-illness psilocybin research. |
| 32 | Bender & Hellerstein (2022) | Clinical review | Second-wave research on classic psychedelics | Safety; risk-benefit evaluation | Clinical risk-benefit framework | Synthesized benefits, risks, and clinical considerations relevant to participant selection and monitoring. |
| 33 | Bouchet et al. (2024) | Systematic review | Older adults represented in PAT trials; multiple compounds | Clinical outcomes; representativeness | Age-related eligibility, outcomes, and safety assessments | Documented the limited representation of older adults and implications for generalizability. |
| 34 | Brennan et al. (2023) | Systematic review | Psychedelic clinical trials; multiple compounds | Therapeutic process; fidelity; reporting | Psychological-support and psychotherapy reporting variables | Identified inconsistent reporting of psychological support, therapy, and psychosocial intervention components. |
| 35 | Driscoll & Lakhan (2025) | Systematic review | Digitally enabled PAT in non-clinical or decentralized settings | Implementation; safety; service delivery | Digital and at-home implementation models | Reviewed safety, efficacy, and implementation issues in digitally enabled or decentralized PAT. |
| 36 | Ferreira & Reis-Pina (2025) | Systematic review | People with life-threatening illnesses; predominantly psilocybin-assisted interventions | Acute subjective, spiritual, and existential outcomes | Mystical-experience, spirituality, quality-of-life, and meaning measures | Synthesized spirituality and mystical-experience outcomes in serious-illness PAT. |
| 37 | Fonseka & Woo (2023) | Literature review | Trauma-related conditions; psilocybin and MDMA | Clinical outcomes; mechanisms | Trauma symptom and related clinical measures | Reviewed therapeutic applications and proposed mechanisms in trauma treatment. |
| 38 | Freitas et al. (2025) | Systematic review | Psilocybin-assisted psychotherapy studies | Safety and adverse events | Adverse-event definitions and monitoring procedures | Documented variability in safety assessment and adverse-event reporting. |
| 39 | Kelly et al. (2023) | Review and perspective | Neurosurgery and neuro-oncology populations and clinical contexts | Clinical and existential outcomes; implementation | Clinical, quality-of-life, and translational assessment considerations | Reviewed opportunities and assessment considerations for psychedelic science in neuro-oncology and neurosurgery. |
| 40 | Korkmaz et al. (2024) | Narrative/systematic review | Depression and substance-use disorders; multiple psychedelic compounds | Clinical outcomes | Depression, substance-use, and related clinical measures | Reviewed evidence and mechanisms for psychedelic therapy in depression and substance-use disorders. |
| 41 | Sabe et al. (2025) | Overview, systematic review, and meta-analysis | Human psychedelic studies; clinical and non-clinical populations | Safety | Psychosis incidence and related psychiatric outcomes | Synthesized evidence concerning psychedelic-induced psychosis and psychiatric risk. |
| 42 | Soto-Angona et al. (2024) | Scoping review | Human studies of classic psychedelics | Safety | Seizure events and seizure-risk factors | Mapped evidence on seizure risk and highlighted limitations of available safety data. |
| 43 | Wong et al. (2025) | Systematic review | Serotonergic psychedelic therapy studies | Biological markers; clinical response | Neurobiological, physiological, and imaging biomarkers | Reviewed biological markers associated with treatment response. |
| 44 | Wall et al. (2023) | Review | Psychedelic drug-development studies; multiple compounds | Biological markers; mechanisms | Neuroimaging methods and biomarkers | Reviewed past, current, and future uses of neuroimaging in psychedelic drug development. |
| 45 | Dominiak et al. (2025) | Umbrella review of meta-analyses | Randomized controlled trials across psychiatric conditions and psychedelic compounds | Clinical outcomes; safety | Cross-indication efficacy and safety outcomes; GRADE assessment | Integrated evidence from meta-analyses and evaluated certainty across indications. |
| 46 | Hultgren et al. (2025) | Meta-analysis | Psilocybin-assisted therapy studies | Therapeutic process; clinical outcomes | Amount of therapy hours and treatment outcomes | Found no statistically significant association between the number of therapy hours and treatment outcomes; highlighted inadequate reporting and lack of standardized therapy protocols. |
| 47 | Earleywine & Kamilar-Britt (2023) | Methodological commentary | Replication studies of PAT | Trial design; statistical interpretation | Prediction intervals and replication expectations | Recommended prediction intervals for interpreting replication evidence. |
| 48 | Feduccia et al. (2023) | Methodological/clinical perspective | Psychedelic medicine across settings and compounds | All phases; standards; implementation | Best-practice and gold-standard recommendations | Argued for standardized best practices, training, procedures, and outcome assessment. |
| 49 | Muthukumaraswamy et al. (2025), corrigendum | Linked corrigendum | Same scope as reference 3 | All phases; trial design | Correction linked to the complex-intervention article | Linked report to reference 3; provides no independent substantive evidence. |
| 50 | Pronovost-Morgan et al. (2025) | International Delphi consensus | Psychedelic clinical trials across compounds and indications | Setting; reporting; implementation | ReSPCT checklist | Produced consensus on 30 extra-pharmacological variables for standardized reporting of setting. |
| 51 | Schruers & Leibold (2025) | Clinical commentary | Psychedelic-assisted psychotherapy | Safety and adverse events | Adverse-event monitoring and reporting considerations | Highlighted the need for systematic monitoring of physiological and psychological adverse events. |
| 52 | Zuljevic et al. (2026) | Cross-sectional methodological study | Registered and published MDMA and psilocybin trials | Safety and outcome reporting | Protocol-publication consistency assessment | Identified inconsistencies between trial registrations and corresponding publications. |
| 53 | Johansen et al. (2022) | Systematic review protocol | Classic psychedelic treatment of depression | Psychological processes and mechanisms | Planned review of psychological process measures | Specified methods for synthesizing psychological mechanisms in psychedelic treatment of depression. |
| 54 | Adkins Singh & de Assis (2025) | Clinical integration framework | Psychedelic integration across cultural and clinical contexts | Post-session integration | THRIVE model | Proposed a holistic integration framework combining indigenous knowledge and evidence-based practice. |
| 55 | Azevedo et al. (2023) | Ethical and conceptual analysis | Psychiatric and broader uses of psychedelics | Ethics; consent; governance | Ethical, legal, social, and clinical framework | Mapped ethical challenges, including consent, vulnerability, professional conduct, and governance. |
| 56 | Hatfield et al. (2024) | Regulatory and policy taxonomy | Australian PAT policy and regulation | Regulation; implementation | Taxonomy of regulatory and policy matters | Organized regulatory and policy issues relevant to PAT implementation. |
| 57 | Hosein et al. (2025) | Multidisciplinary consensus statement | Routine clinical care involving psilocybin | Implementation; safety; service evaluation | Clinical implementation considerations and cautions | Identified requirements and gaps for responsible integration of psilocybin into routine care. |
| 58 | Wolff et al. (2025) | Competency study/framework | Real-world mental-health professionals providing psychedelic treatment | Therapeutic process; workforce competence | Core practitioner competencies | Identified competencies needed for psychedelic treatment in routine mental-health settings. |
| 59 | Yong et al. (2025) | Clinical practice guideline | People with PTSD; MDMA-assisted psychotherapy in Australia | Preparation, dosing, integration; safety; implementation | Australian clinical practice guideline | Developed practice recommendations for MDMA-assisted psychotherapy for PTSD. |
| 60 | Bender et al. (2025) | Empirical provider-perspective study | Providers involved in psychedelic therapy | Therapeutic process; implementation | Provider-reported treatment challenges | Documented practical and clinical challenges encountered during psychedelic therapy. |
| 61 | Lewis et al. (2023) | Pilot clinical study | Patients with cancer; psilocybin-enhanced group psychotherapy | Clinical and existential outcomes; group process | Psychiatric, quality-of-life, and group-therapy assessments | Examined feasibility and outcomes of psilocybin-enhanced group psychotherapy in cancer care. |
| 62 | Luoma et al. (2024) | Instrument-development / empirical study | MDMA-assisted therapy context | Therapeutic process | Self-report measure of therapeutic touch | Highlighted touch as a process variable and introduced a measure for its assessment. |
